# Genetic polymorphisms of BDNF on cognitive functions in drug-naive first episode patients with schizophrenia

**DOI:** 10.1038/s41598-021-99510-7

**Published:** 2021-10-08

**Authors:** Xiuru Su, Limin Qiao, Qing Liu, Yujie Shang, Xiaoni Guan, Meihong Xiu, Xiangyang Zhang

**Affiliations:** 1 Department of Psychiatry, Hebei Province Veterans Hospital, Baoding, People’s Republic of China; 2 Lian Yang Community Health Service Center, Shanghai, People’s Republic of China; 3 Qingdao Mental Health Center, Qingdao University, Qingdao, People’s Republic of China; 4 Peking University HuiLongGuan Clinical Medical School, Beijing HuiLongGuan Hospital, Changping District, Beijing, 100096 People’s Republic of China; 5 CAS Key Laboratory of Mental Health, Institute of Psychology, Chinese Academy of Sciences, 16 Lincui Road, Chaoyang District, Beijing, 100101 People’s Republic of China

**Keywords:** Psychiatric disorders, Genotyping and haplotyping

## Abstract

Brain-derived neurotrophic factor (BDNF) is reported to be involved in cognitive decline in patients with schizophrenia (SZ). Previous studies have found that cognitive deficits remain stable during the chronic disease phase in SZ, but the findings were inconsistent. The role of BDNF in cognitive deficits at different stage of illness remains unclear. This study aimed to examine the effect of *BDNF* polymorphisms on cognitive deficits in drug-naïve first-episode (DNFE) patients and chronic patients with SZ. 262 DNFE patients, 844 chronic patients, and 1043 healthy controls were recruited to compare 4 polymorphisms in *BDNF* gene and cognitive function. We found that there was no significant difference in genotype and allele frequencies between SZ patients and controls. However, they were closely related to cognitive functioning. *BDNF* rs2030324 polymorphism played a strong role in language performance only in DNFE patients with SZ. The language index of DNFE patients with rs2030324 TT and TC genotypes was worse than that of chronic patients, but there was no significant difference in CC genotypes between DNFE and chronic patients. Rs6265 had no significant effect on cognitive functioning in patients and controls. Our result suggests *BDNF* gene polymorphisms were related to different domains of cognitive function at the different stage of SZ, especially language in DNFE patients.

## Introduction

Schizophrenia (SZ) is a complex psychiatric disorder with a prevalence rate of about 1%. Cognitive deficits are core symptoms of SZ patients. Patients with SZ show multiple domains of cognitive deficits, such as working memory, attention and executive function^1^. Cognitive deficit occurs in the early stage of SZ and usually persists at all stages of this disorder, and has been shown to be related to the functional disability of patients^2^.

The pathogenesis of SZ is related to the complex interaction between genetic and environmental risk factors at the critical early stage of neurodevelopment. Several hypotheses of neurobiological mechanism have been proposed to explain the pathogenesis of SZ and related neurocognitive phenotype, such as the neurotrophin hypothesis. Brain-derived neurotrophic factor (BDNF) is one of the most widely expressed neurotrophins, which regulates the viability and functional integrity of neuron, mediates the neuroplasticity processes and prevents neuronal damage and degeneration^3^. BDNF is expressed in the early development of life. The complexity of gene structure determines the diversity of its function in the central nervous system (CNS)^4^. Different regulation of promoter results in the production of at least eight 5′ exons and one 3′ exon and these *BDNF* transcripts are widely distributed throughout the CNS with high specificity^5^. *BDNF* gene is originally translated into the precursor BDNF protein, and then is cleaved to produce mature BDNF, which is transported to postsynaptic dendrites or presynaptic terminals^6^. Mature and pro-BDNF interacts with TrkB and p75NTR receptors respectively, which have substantially opposite functions in CNS^7^. Studies support that the concentration of BDNF in blood is related to the expression level of BDNF in brain^8^. Recent meta-analyses reported a decrease in serum or plasma BDNF levels in drug-naïve first-episode (DNFE) patients with SZ^9,10^. In particular, decreased serum level of BDNF has been revealed to be associated with neurocognitive deficits in SZ patients^11,12^.

Single nucleotide polymorphisms (SNPs) in the *BDNF* gene, such as rs6265, rs12273539, rs10835210, and rs2030324, have previously been thought to affect different psychiatric disorders and/or serum levels of BDNF in chronic patients with SZ^13–16^. However, conflicting findings have been observed. In recent reviews and meta-analyses, the association between *BDNF* SNPs and SZ is weak, but the relationship between *BDNF* SNPs and cognitive dysfunction in chronic SZ patients is robust, especially rs6265. This SNP is located in exon IX, leading to a valine-methionine alteration in proBDNF protein, thereby impairing the activity-dependent secretion of mature BDNF^17^. Previous studies found that, compared with SZ patients with non-Met alleles, patients with Met alleles exhibited reduced hippocampal function when performing declarative memory tasks, and less gray matter in the hippocampus and prefrontal lobe^18,19^. Ho et al. found that *BDNF* rs6265 variant was associated with neuroplasticity and longitudinal progressive changes in brain volume^20,21^. The other 3 SNPs (rs12273539, rs10835210, and rs2030324) in the *BDNF* gene have previously found to be associated with SZ^20^. For example, a rs12273539 variant or haplotype containing this SNP allele was found to be correlated with major depressive disorder^14^. Moreover, rs10835210 and rs2030324 were also found to increase the risk of SZ^16,22^. In particular, the haplotypes of these 4 SNPs were closely related to the clinical symptoms and cognitive dysfunction in patients with chronic SZ^23,24^. Therefore, in this study, we selected these four SNPs to examine their relationship with the cognitive function of DNFE patients and chronic patients.

Cognitive deficits are present across all phases of SZ, from early to more chronic disease, with defects in several dimensions in the whole course of the disease. Previous studies support that various BDNF polymorphisms were related to the impairments of different domains of cognitive functions. For example, SZ patients who are Met allele carrier had lower hippocampal function and performed worse in a declarative memory task than those who are not Met allele carriers^18^. For SZ patients who were heterozygous carriers of rs12273539, their performance in attention index and RBANS total scores was consistently better than that of T/T mutation homozygous patients^23^. In addition, rs10835210 has shown a critical role in language performance of SZ patients^23^. Considering that most previous studies have investigated the influence of *BDNF* SNPs on cognition during the onset or chronic phase of the disease, and did not fully map the course of the disease, our current study was the first to investigate the role of *BDNF* gene in the cognitive deficits of a large sample of chronic patients and DNFE patients. We hypothesized that various *BDNF* SNPs may have different effects on the neurocognitive function of DNFE SZ, chronic SZ and HCs. Therefore, we were to examine the possible role of these 4 SNPs in neurocognitive deficits of patients with DNFE and chronic SZ.

## Subjects and methods

### Subjects

The study included 262 DNFE patients with SZ recruited from Beijing Huilongguan Hospital and Henan Zhumadian Hospital. Diagnosis was made by experienced psychiatrists according to the Chinese version of the Structured Clinical Interview for DSM-IV (SCID) at admission^25^. Inclusion criteria were: aged of 18–45 years, disease duration < 5 years, antipsychotics free or a cumulative antipsychotic treatment < 2 weeks, without physical diseases (i.e. diabetes, hypertension, hyperthyroidism and cancer). In this study, we defined the patients as DNFE as a prior study by Lieberman et al.^26^. 844 inpatients were recruited from Beijing Huilongguan Hospital and Hebei Province Veterans Hospital. Inclusion criteria were: aged of 25–75 years, disease duration > 5 years, receipt of stable doses of antipsychotics for at least 6 months. 1043 unrelated healthy subjects were recruited in the local community. Experienced clinical psychiatrists ruled out Axis I disorders by using SCID.

The severity of symptom was assessed by six experienced psychiatrists by using the PANSS^27^. Cognitive functioning was rated by using the Repeatable Battery for the Assessment of Neuropsychological Status (RBANS, Form A), as reported in our previous study^1^. The RBANS comprises 5 index scores and a total score. Test indices are immediate memory, visuospatial/constructional, language, attention, and delayed memory.

The current study was conducted in accordance with the Declaration of Helsinki and approved by the Institutional Review Board of Beijing Huilongguan Hospital. All subjects signed the informed consent before entry into the study.

### Genotyping

Whole blood DNA for each participant was extracted using the Wizard Genomic DNA purification kit (Promega, USA). Restriction fragment length polymorphism (RFLP) method was used to genotype the 4 SNPs in *BDNF* gene as described in our previous study^23^. Genotyping was duplicated by 2 investigators independently for accuracy and carried out with the investigators blind to the clinical status. If the genotypic assignment of the 2 researchers was inconsistent, the sample is repeated. In addition, genotyping error check was conducted by re-genotyping of a sub-sample (n = 50). The reproducibility was usually more than 0.99.

### Statistical analysis

ANOVA and *χ*^2^ test were carried out to explore whether there was difference in demographic data and clinical data between patients and HCs. *χ*^2^ goodness-of-fit test was performed to examine the deviation from the Hardy–Weinberg equilibrium (HWE) separately in patients and HCs. *χ*^2^ test was conducted to analyze the difference in the allele and genotype frequencies for the polymorphisms between patients and HCs.

The MANCOVA in the general linear model was conducted to investigate the relationship between *BDNF* genotypes and cognitive measures. For the main models, diagnostic group (DNFE vs chronic SZ vs HC) and genotypes were entered as independent variables. Cognitive index scores and RBANS total score were entered as the dependent variables, with sex, age and education as covariates. In the models, the main effects of diagnosis and genotype and the interaction effect of diagnosis × genotype were examined. The genotype × diagnosis interaction in the models detects any potential differential effect of alleles on the cognitive function between diagnostic groups. Further multiple regression analysis used the RBANS scores as dependent variable, and the genotype group (the number of variant alleles, such as 0, 1, or 2 alleles) as the independent variables in patients or HCs, respectively. For HCs, the covariates in the model included sex, age, and education. For patients, the covariates in the models included sex, age, education, onset age and PANSS scores.

The PASW Statistics 20.0 was used to conduct all statistical analysis. All p values were 2-tailed at the significance level of *p* < 0.05. Haploview 4.2 (Daly Laboratory Inc, Cambridge, Massachusetts) was used to compute the pairwise linkage disequilibrium (LD), haplotype block and haplotype frequency in patients and controls. Rare haplotypes found in less than 1% were excluded from association analysis. 10,000 permutations were conducted for the most significant test to determine the empirical significance. Since we had the RBANS total score and its five subscores among three genotypes of 4 SNPs in *BDNF* gene, the Bonferroni-adjusted significance level was set at *p* = 0.05/72 = 0.00069.

## Results

Demographic and clinical data between DNFE patients, chronic patients and HCs were shown in Table 1. Significant differences were observed in sex and age between DNFE patients, chronic patients and HCs (both *p* < 0.05).

**Table 1:** Demographic, clinical data and neurocognitive function in SZ patients and HCs.

Clinical variable	DNFE SZ	Chronic SZ	HCs	F or *χ*^*2*^	*P* value
	(n = 262)	(n = 844)	(n = 1043)		
Sex(male/female), n	150/112	663/181	586/457	110.8	< 0.001
Age, y	26.8 ± 9.0	47.8 ± 9.7	44.9 ± 13.6	36.8	< 0.001
Education, y	9.2 ± 3.8	8.9 ± 2.7	9.5 ± 3.3	1.6	0.43
Age at onset, y	26.0 ± 9.5	23.5 ± 5.4		5.4	0.001
No. of hospitalizations		4.2 ± 2.6			
**Antipsychotic types, n (%)**					
Typical		182 (22)			
Atypical		663 (78)			
Antipsychotic dose, mg/days (CPZ equivalents)		466 ± 429			
PANSS total score	75.5 ± 17.2	59.8 ± 15.8		5.8	0.002
P subscore	21.6 ± 6.3	11.7 ± 5.2		8.7	< 0.001
N subscore	19.0 ± 7.0	22.8 ± 8.4		1.5	0.21
G subscore	35.3 ± 9.5	25.2 ± 6.4		5.1	0.001
**RBANS**					
Immediate memory	65.3 ± 16.7	58.8 ± 16.4	75.6 ± 17.6	135.3	< 0.001
VC	77.3 ± 17.0	78.1 ± 19.0	80.0 ± 15.4	1.8	0.16
Language	75.1 ± 18.6	81.7 ± 15.7	94.1 ± 13.2	140.4	< 0.001
Attention	74.4 ± 19.8	71.1 ± 17.8	87.5 ± 20.0	104.5	< 0.001
Delayed memory	70.1 ± 20.0	67.3 ± 19.4	86.5 ± 15.0	156.6	< 0.001
Total score	66.6 ± 15.9	65.0 ± 15.3	80.2 ± 15.0	140.7	< 0.001

Abbreviations: *DNFE* drug-naïve and first episode, *SZ* schizophrenia, *HC* healthy controls, *CPZ* chlorpromazine, *P* positive symptom, *N* negative symptom, *G* general psychopathological symptom, *VC* visuospatial/constructional, *RBANS* Repeatable Battery for the Assessment of Neuropsychological Status.

### Allele, genotype frequencies and LD analysis of four BDNF SNPs between patients and controls

Table 2 showed the genotype and allele frequencies of 4 SNPs in *BDNF* gene. No deviation from HWE was observed in controls or patients (all *p* > 0.05). There was no significant difference in the allele and genotype frequencies between SZ and HC (all *p* > 0.05) (Table 2).

**Table 2:** Genotypic and allelic distributions for SNPs in the *BDNF* gene between SZ patients and HCs.

Marker	M/m	Group	Genotype					Allele			
			M/M	M/m	m/m	*χ*^*2*^	*p*	M	m	*χ*^*2*^	*p*
rs6265	G/A	DNFE	60	142	60	2.0	0.36	262	262	0.001	0.97
		Chronic	211	456	177			878	810		
		HC	271	532	240			1074	1012		
rs12273539	T/C	DNFE	172	85	5	1.3	0.53	429	95	0.04	0.85
		Chronic	582	236	25			1400	286		
		HC	709	313	21			1731	355		
rs10835210	C/A	DNFE	137	110	15	5.0	0.08	384	140	4.7	0.03
		Chronic	397	371	76			1165	523		
		HC	553	417	73			1523	563		
rs2030324	C/T	DNFE	63	140	59	2.9	0.23	266	258	2.4	0.12
		Chronic	279	430	135			988	700		
		HC	344	542	156			1230	854		

Abbreviations: *m* minor allele, *M* major allele.

LD analyses were conducted on all polymorphism pairs in both patients and healthy controls. The four polymorphisms were in modest LD with each other in both the control and the case groups (D′ = 0.72–0.94, r^2^ = 0.04–0.48) (Supplement Figure 1).

### Cognition in DNFE, chronic patients and HCs

Cognition data were available from 243 DNFE patients, 261 chronic patients and 434 HCs. After controlling for age, sex and education, decreased RBANS total score and its 5 index scores were observed in chronic patients compared to DNFE patients (all *p* < 0.05). In addition, RBANS scores were lower in the combined with DNFE and chronic patients group as compared to HCs (all *p* < 0.05).

### Genotype effects on cognitive performance in patients and HCs

As shown in Supplement Table 1, the separate MANCOVA analysis including DNFE patients and HCs showed a significant main effect of rs12273539 on visuospatial/construction index and significant diagnosis-by-genotype effects on visuospatial/construction index and RBANS total score (all *p* < 0.05). Moreover, there was a significant main effect of rs2030324 on attention (F = 4.1, *p* = 0.017), language (F = 5.5, *p* = 0.004) and RBANS total score (F = 3.6, *p* = 0.028). Also, there were significant diagnosis-by-genotype effects on language index (F = 8.2, *p* < 0.001) and visuospatial/construction index (F = 3.7, *p* = 0.026). However, only the diagnosis-by-genotype effect on language remains significant after Bonferroni correction (*p*_Bonferroni corrected_ < 0.05). Post hoc test found that there were significant differences between the T/T versus C/T versus C/C in language in patients (*p* < 0.05), but not in HCs (*p* = 0.59). Further multiple regression analysis inclusion of heterozygotes, education, age and sex as the independent variables revealed significant linear relationship between the language index score and the number of T variant alleles (eg, 2, 1, or 0 alleles) within DNFE patients (*p* = 0.011), but not in HCs. *BDNF* rs2030324 genotype only accounted for 7.6% of the variance in language index score in DNFE patients, indicating a weak effect of the variant T allele of the rs2030324 polymorphism on language in SZ.

For the chronic patients and HCs, there was a significant main effect of rs12273539 on language index score, attention index score and RBANS total score (all *p* < 0.05). Moreover, we found a significant diagnosis-by-rs10835210 effect on immediate memory (*p* < 0.01). However, all these differences were not significant after Bonferroni correction (all *p*_Bonferroni corrected_ > 0.05).

For the DNFE and chronic patients, the MANCOVA analysis showed significant main effects of rs12273539 on visuospatial/construction index (F = 4.0, *p* = 0.018) and RBANS total score (F = 3.2, *p* = 0.04). There was a significant main effect of rs10835210 on immediate memory (F = 3.2, *p* = 0.04) and delayed memory (F = 3.6, *p* = 0.03) and a significant diagnosis-by-rs2030324 interaction effect on language index (F = 3.6, *p* = 0.03). Moreover, we also found a significant diagnosis-by-rs2030324 interaction effect on language index (F = 8.2, *p* < 0.001), attention index (F = 5.6, *p* = 0.004), immediate memory (F = 4.1, *p* = 0.017) and RBANS total score (F = 4.1, *p* = 0.018). After Bonferroni correction, only the interaction effect of diagnosis-by-rs2030324 on language index remained significant (*p*_Bonferroni corrected_ < 0.05). Our further analysis revealed that DNFE patients with rs2030324 TT and TC genotype performed worse in language index score, compared to those in chronic patients (*p* < 0.01). There was no difference in language index between patients with CC genotype in early stage (DNFE patients) and chronic stages of the illness (chronic patients) (*p* > 0.05).

In addition, we found no association of rs6265 with cognitive functions in patients and controls (all *p* > 0.05).

## Discussion

The main results of the current study were as follows. First, compared with HCs, cognitive function was lower in DNFE patients. In addition, cognitive function was lower in chronic patients than in DNFE patients. Second, rs2030324 variant was closely related to multiple domains of neurocognitive impairment in DNFE patients, but not in HCs and chronic patients. Third, the language index of DNFE patients with rs2030324 TT and TC genotypes was worse than that of chronic patients, but there was no significant difference in CC genotypes between DNFE and chronic patients.

We found that several domains of cognitive dysfunction assessed by RBANS were impaired in DNFE patients and chronic patients with SZ as compared to HCs. Moreover, in this large sample study, we also found that cognitive function was more seriously impaired in chronic patients than DNFE patients. Our findings suggest that multiple dimensions of cognitive deficits exist in the early onset of psychosis and deteriorate further during the chronic progressive course of SZ patients. These results were consistent with other studies^28,29^. For example, a previous study reported that verbal knowledge and memory decreased significantly, while processing speed and executive functions remained unchanged after 10 years follow-up since the first episode, supporting our findings that cognitive decline varied across cognitive dimensions^30^. However, our finding was inconsistent with a 15-year follow-up study, which reported that cognitive deficit is already present at the onset of SZ and remains stable over the follow-up period^28^. The difference between Albus et al. and current study may be caused by the recruitment of patients (SZ, schizophreniform disorder, schizoaffective disorder vs SZ), age (below 60 years vs below 45 years) and the use of different cognitive scales (several independent cognitive tests vs RBANS scale).

Our further finding showed that there was no association between *BDNF* 4 tag SNPs and SZ, which was in line with a recent genome-wide association studies (GWAS) based hypothesis-driven candidate gene^31^. Our study with the large sample provides further evidence that *BDNF* gene is not a risk gene for SZ. Notably, we found a positive association between *BDNF* polymorphisms and cognition. Rs12273539 variant was shown to be closely related to neurocognitive deficits measured by RBANS in HCs, DNFE and chronic patients, indicating that *BDNF* rs12273539 polymorphism may play a fundamental role in human neurocognitive function in both SZ patients and HCs. However, the effects of rs12273539 on cognitive functions are not significant after Bonferroni correction, suggesting that it is a weak effect.

Interestingly, we found that, while rs2030324 variant affects several cognitive dimensions only in DNFE patients, but not in HCs, suggesting a crucial role of *BDNF* gene in the early stage SZ. Specifically, compared with chronic patients with rs2030324 TT and TC genotype, DNFE patients with TT and TC genotype performed worse in several areas of cognitive function, such as language, attention and RBANS total score. However, after Bonferroni correction, only the interaction effect on language remained significant. The language index score of CC carriers was not different between DNFE patients and chronic patients. The mechanisms by which this variant produced differential effects on cognition in DNFE patients than in chronic patients remain unclear. We speculated that the underlying mechanism was related to a possible influence of rs2030324 variant on the levels of serum BDNF. Rs2030324 variant is located in the promoter of *BDNF* gene and it may down-regulate the levels of BDNF in the CNS^16^. T allele in rs2030324 variant may be associated with a lower level of BDNF, which further influence the structural and functional plasticity of the hippocampus. In contrast, the above association was not observed in chronic patients after treatment with long-term antipsychotics. Studies reported that antipsychotics can regulate the levels of BDNF^32–34^, which could provide an explanation for the finding of no relationship between rs2030324 variant and cognitive deficits in chronic patients. However, this is only our speculation. It is still unclear whether rs2030324 regulates serum BDNF levels or activity. Another possible mechanism involves the complex interaction between rs2030324 and other SNP in *BDNF* gene, especially the key polymorphism influencing cognitive performance, namely rs6265. Rs6265 is a functional SNP affecting the activity-dependent release of BDNF. Previous studies have reported that rs6265 Met allele carriers performed worse in visuospatial/construction and episodic memory in both HCs and SZ patients^18^. Contrary to our expectation, we did not confirm the role for rs6265 polymorphism in cognitive functioning among HCs or individuals with SZ in our present study. Therefore, we could not explain why the functional effects of the *BDNF* rs2030324 variant produced different effects on cognitive function in DNFE patients than in chronic patients. A future study including a large sample with a longitudinal design to investigate whether the *BDNF* polymorphisms affect circulating levels of BDNF protein and cognitive function is warranted.

It is worthy of mentioning that the assessment of cognition is state dependent and is influenced by moderators, such as patient type (first episode vs chronic illness, active phase vs remission), disease duration, treatment history, and the different cognitive tests. The difference in those confounding factors might be the cause of the cognitive functions across genotypes. To minimize those confounding factors, we evaluated the RBANS scale with the same raters in HC subjects and patients. In addition, all participants were recruited in the same period. However, we did not control other confounding variables that affect cognitive functioning, such as depression, social isolation, medication compliance in the course of chronic illness, or physical health problems such as obesity, diabetes and cerebrovascular disease. Further, although we have controlled for age and education in all statistical analyses, chronic patients were older and less educated than DNFE patients in this study, which may be the reason for the group differences. In considering these, we further compared chronic patients with younger age and higher educational levels with DNFE patients, and the results still supported our findings.

Several limitations should be noted here. First, although our present study sample is relatively large, the sample size is still small because of the expected small effects of genetic variation. In particular, a lot of subjects only have been genotyped, but not been assessed for cognitive functions. Second, we compared the cognition between DNFE patients and chronic patients, however, we did not compare the cognitive function of patients at the first episode of psychosis and at follow-up after treatment with antipsychotics. A future longitudinal study could better reveal the effects of *BDNF* polymorphisms or the chronic disease progression on cognition. Third, we did not investigate the effects of *BDNF* polymorphisms on a neural functional network for the cognition of the participants by magnetic resonance imaging (MRI), therefore, we cannot give a causative inference to explain whether the SNPs in *BDNF* gene influence the critical neural circuit, which in turn affect cognitive function in SZ patients. Fourth, previous studies have shown that different antipsychotic drugs may have different effects on the cognitive functions of chronic SZ^35,36^. Therefore, our findings in in the cognitive function differences of the four BDNF SNPs in this study may be due to different antipsychotics, although we controlled for them as confounding factors in all these analyses.

Overall, we found that cognitive deficit occurs in the first stage and some domains are further weakened with disease progression. *BDNF* rs12273539 polymorphism was closely related to cognition in both patients and HCs. Interestingly, rs2030324 was associated with cognitive deficits only in DNFE patients, but not in HCs or chronic patients, suggesting that rs2030324 variant plays a critical role in the pathophysiology in the early stage of SZ. In addition, the language performance of rs2030324 TT and TC carriers in AFNE patients is worse than that in chronic patients, but no difference was found in CC carriers. Our findings are of great significance in understanding the role of *BDNF* polymorphisms in the different domains of cognitive deficits in SZ patients at different stages.

## Supplementary Information

Supplementary Figure 1.

Supplementary Table 1.
