# Supplementary material for: Genetic polymorphisms of BDNF on cognitive functions in drug-naive first episode patients with schizophrenia
Source: Sci Rep. 2021 Oct 8;11:20057. doi: 10.1038/s41598-021-99510-7 (PMC8501135; doi:10.1038/s41598-021-99510-7)
Supplement: Supplementary file 1 — Supplementary Figure 1. [file 41598_2021_99510_MOESM1_ESM.doc]

**Patients with schizophrenia Healthy controls**


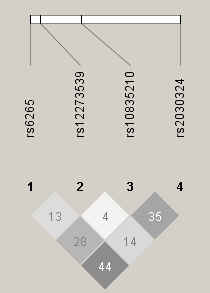

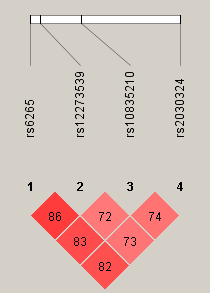

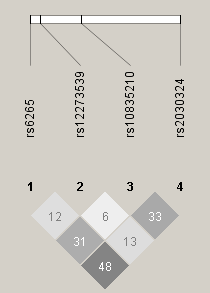

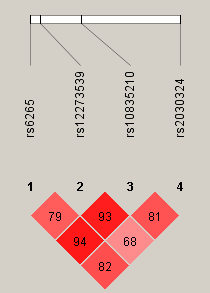


**Figure 1.** Genomic structure of BDNF, including relative location of 4 SNPs studied and linkage disequilibrium (LD) of these 4 SNPs in the patients with schizophrenia and control groups. The LD between pairwise SNPs, using D’ (red color) and r^2^ (grey color) values, are shown separately for cases and controls. High levels of LD are represented by increasing scale intensity from 0 to 100, as shown by the bars.
