# Supplementary material for: Genetic polymorphisms of BDNF on cognitive functions in drug-naive first episode patients with schizophrenia
Source: Sci Rep. 2021 Oct 8;11:20057. doi: 10.1038/s41598-021-99510-7 (PMC8501135; doi:10.1038/s41598-021-99510-7)
Supplement: Supplementary file 2 — Supplementary Table 1. [file 41598_2021_99510_MOESM2_ESM.doc]

**Table 1 Comparisons of total and index scores on the RBANS by diagnostic and genotype groups^a^**

| Genotype | DNFE SZ | | | HCs | | | Genotype  p value | Genotype × Diagnosis, |
| --- | --- | --- | --- | --- | --- | --- | --- | --- |
|  | M/M | M/m | m/m | M/M | M/m | m/m | (adjusted p) | p value |
|  | n=57 | n=131 | n=55 | n=138 | n=207 | n=73 |  | (adjusted p) |
| *rs2030324* |  |  |  |  |  |  |  |  |
| VC | 81.8±19.9 | 78.4±16.4 | 71.0±12.7 | 78.7±15.8 | 80.6±15.0 | 80.0±16.2 | 0.14(>0.05) | 0.026(>0.05) |
| IM | 70.0±15.6 | 65.1±17.2 | 61.4±15.4 | 76.4±17.1 | 75.4±17.2 | 74.9±18.1 | 0.14(>0.05) | 0.11(>0.05) |
| DM | 73.2±20.1 | 69.3±20.4 | 69.5±18.9 | 86.4±15.8 | 87.7±14.4 | 83.9±14.3 | 0.82(>0.05) | 0.95(>0.05) |
| Language | 82.9±15.8 | 74.9±18.8 | 67.9±17.7 | 93.9±12.7 | 94.2±13.8 | 94.6±12.3 | 0.004(>0.05) | <0.001(<0.05) |
| Attention | 81.3±20.6 | 73.9±19.3 | 68.2±17.8 | 88.3±21.0 | 87.9±19.9 | 84.1±19.0 | 0.017(>0.05) | 0.22(>0.05) |
| Total score | 72.5±17.0 | 66.5±15.6 | 61.1±12.7 | 80.3±15.1 | 80.7±14.8 | 78.7±15.0 | 0.025(>0.05) | 0.07(>0.05) |
|  | M/M | M/m | m/m | M/M | M/m | m/m |  |  |
|  | n=158 | n=76 | n=4 | n=290 | n=134 | n=9 |  |  |
| *rs12273539* |  |  |  |  |  |  |  |  |
| VC | 77.0±16.5 | 76.7±16.1 | 102.0±34.7 | 78.8±15.8 | 81.6±14.8 | 80.1±12.7 | 0.024(>0.05) | 0.03(>0.05) |
| IM | 65.1±16.0 | 65.1±17.5 | 76.0±26.3 | 74.6±17.6 | 77.9±16.8 | 67.8±12.4 | 0.82(>0.05) | 0.12(>0.05) |
| DM | 70.9±19.9 | 67.9±19.8 | 80.3±31.0 | 85.8±15.8 | 87.8±13.6 | 81.1±11.4 | 0.69(>0.05) | 0.21(>0.05) |
| Language | 75.6±18.6 | 73.6±18.6 | 83.5±24.3 | 92.8±13.4 | 97.2±12.0 | 84.2±16.6 | 0.66(>0.05) | 0.05(>0.05) |
| Attention | 73.9±20.1 | 74.9±18.8 | 84.3±30.0 | 85.5±20.4 | 91.3±19.1 | 90.2±17.8 | 0.17(>0.05) | 0.52(>0.05) |
| Total score | 66.5±15.5 | 65.9±15.7 | 83.8±28.2 | 78.9±15.3 | 83.0±14.0 | 72.9±10.2 | 0.39(>0.05) | 0.007(>0.05) |
|  | M/M | M/m | m/m | M/M | M/m | m/m |  |  |
|  | n=126 | n=99 | n=14 | n=229 | n=177 | n=24 |  |  |
| *rs10835210* |  |  |  |  |  |  |  |  |
| VC | 76.7±17.5 | 79.3±17.0 | 71.1±13.1 | 79.2±15.2 | 80.2±15.8 | 81.6±15.8 | 0.35(>0.05) | 0.25(>0.05) |
| IM | 64.3±17.0 | 66.8±16.7 | 64.3±14.4 | 76.7±17.5 | 73.4±16.7 | 76.5±19.9 | 0.73(>0.05) | 0.41(>0.05) |
| DM | 70.0±20.3 | 71.5±19.6 | 62.4±20.4 | 86.1±15.7 | 86.4±14.0 | 85.5±17.1 | 0.41(>0.05) | 0.48(>0.05) |
| Language | 73.1±19.8 | 76.6±17.3 | 82.7±13.8 | 94.6±13.3 | 93.1±13.0 | 94.5±12.2 | 0.15(>0.05) | 0.10(>0.05) |
| Attention | 73.8±20.0 | 75.4±19.8 | 75.3±21.6 | 89.1±20.1 | 84.7±20.2 | 87.9±17.3 | 0.32(>0.05) | 0.57(>0.05) |
| Total score | 65.9±16.7 | 68.0±15.3 | 64.6±13.3 | 80.6±14.8 | 78.9±15.0 | 80.7±15.5 | .97(>0.05) | 0.65(>0.05) |
|  | M/M | M/m | m/m | M/M | M/m | m/m |  |  |
|  | n=158 | n=76 | n=4 | n=167 | n=84 | n=7 |  |  |
| *rs6265* |  |  |  |  |  |  |  |  |
| VC | 77.9±18.5 | 77.2±16.5 | 77.7±16.5 | 81.7±15.0 | 79.5±15.3 | 77.7±16.3 | 0.39(>0.05) | 0.55(>0.05) |
| IM | 66.6±16.4 | 65.2±17.3 | 64.6±15.3 | 76.1±16.5 | 75.0±18.1 | 75.6±16.5 | 0.70(>0.05) | 0.95(>0.05) |
| DM | 68.2±19.7 | 70.1±20.3 | 72.8±19.6 | 86.1±14.3 | 86.9±14.9 | 85.5±16.4 | 0.48(>0.05) | 0.27(>0.05) |
| Language | 78.4±16.1 | 74.4±19.2 | 73.6±19.3 | 93.8±12.7 | 94.7±13.4 | 93.0±13.4 | 0.18(>0.05) | 0.24(>0.05) |
| Attention | 74.8±20.2 | 74.8±19.0 | 72.7±21.3 | 87.0±19.3 | 87.7±20.3 | 86.8±20.8 | 0.43(>0.05) | 0.81(>0.05) |
| Total score | 67.3±15.8 | 66.6±15.6 | 66.3±16.5 | 80.5±14.5 | 80.3±15.3 | 78.8±14.8 | 0.52(>0.05) | 0.74(>0.05) |
|  | Chronic SZ | | | HCs | | |  |  |
|  | M/M | M/m | m/m | M/M | M/m | m/m |  |  |
|  | n=167 | n=84 | n=7 | n=290 | n=134 | n=9 |  |  |
| *rs12273539* |  |  |  |  |  |  |  |  |
| VC | 83.7±18.5 | 84.9±18.5 | 90.4±22.5 | 78.8±15.8 | 81.6±14.8 | 80.1±12.7 | 0.27(>0.05) | 0.66(>0.05) |
| IM | 64.3±19.1 | 64.5±19.2 | 70.4±20.6 | 74.6±17.6 | 77.9±16.8 | 67.8±12.4 | 0.50(>0.05) | 0.19(>0.05) |
| DM | 70.7±20.3 | 74.4±21.4 | 76.7±25.6 | 85.8±15.8 | 87.8±13.6 | 81.1±11.4 | 0.17(>0.05) | 0.44(>0.05) |
| Language | 86.3±14.2 | 89.0±13.8 | 91.9±6.5 | 92.8±13.4 | 97.2±12.0 | 84.2±16.6 | 0.006(>0.05) | 0.07(>0.05) |
| Attention | 80.9±15.6 | 84.4±15.1 | 85.9±16.5 | 85.5±20.4 | 91.3±19.1 | 90.2±17.8 | 0.01(>0.05) | 0.76(>0.05) |
| Total score | 71.3±15.0 | 74.1±16.0 | 78.9±16.1 | 78.9±15.3 | 83.0±14.0 | 72.9±10.2 | 0.04(>0.05) | 0.16(>0.05) |
|  | M/M | M/m | m/m | M/M | M/m | m/m |  |  |
|  | n=130 | n=111 | n=20 | n=229 | n=177 | n=24 |  |  |
| *rs10835210* |  |  |  |  |  |  |  |  |
| VC | 84.1±18.1 | 84.5±17.6 | 76.7±24.7 | 79.2±15.2 | 80.2±15.8 | 81.6±15.8 | 0.49(>0.05) | 0.18(>0.05) |
| IM | 63.1±17.7 | 67.5±10.9 | 55.4±15.3 | 76.7±17.5 | 73.4±16.7 | 76.5±19.9 | 0.31(>0.05) | 0.005(>0.05) |
| DM | 70.4±20.8 | 75.2±20.7 | 62.7±19.1 | 86.1±15.7 | 86.4±14.0 | 85.5±17.1 | 0.05(>0.05) | 0.07(>0.05) |
| Language | 88.0±13.5 | 87.3±14.2 | 81.0±13.4 | 94.6±13.3 | 93.1±13.0 | 94.5±12.2 | 0.21(>0.05) | 0.21(>0.05) |
| Attention | 81.5±15.3 | 83.0±15.9 | 77.8±16.9 | 89.1±20.1 | 84.7±20.2 | 87.9±17.3 | 0.53(>0.05) | 0.11(>0.05) |
| Total score | 71.6±14.7 | 74.3±16.1 | 64.1±14.9 | 80.6±14.8 | 78.9±15.0 | 80.7±15.5 | 0.24(>0.05) | 0.05(>0.05) |
|  | M/M | M/m | m/m | M/M | M/m | m/m |  |  |
|  | n=67 | n=126 | n=62 | n=108 | n=226 | n=98 |  |  |
| *rs6265* |  |  |  |  |  |  |  |  |
| VC | 82.0±19.5 | 84.4±18.4 | 85.9±17.2 | 81.7±15.0 | 79.5±15.3 | 77.7±16.3 | 0.99(>0.05) | 0.10(>0.05) |
| IM | 63.7±18.3 | 64.5±19.9 | 65.7±18.2 | 76.1±16.5 | 75.0±18.1 | 75.6±16.5 | 0.87(>0.05) | 0.80(>0.05) |
| DM | 71.4±21.2 | 71.9±21.0 | 73.2±20.0 | 86.1±14.3 | 86.9±14.9 | 85.5±16.4 | 0.92(>0.05) | 0.72(>0.05) |
| Language | 87.8±15.5 | 86.1±13.1 | 90.0±13.2 | 93.8±12.7 | 94.7±13.4 | 93.0±13.4 | 0.75(>0.05) | 0.11(>0.05) |
| Attention | 82.2±14.0 | 82.1±14.7 | 82.8±15.7 | 87.0±19.3 | 87.7±20.3 | 86.8±20.8 | 0.99(>0.05) | 0.90(>0.05) |
| Total score | 71.8±15.3 | 72.1±15.8 | 73.7±14.4 | 80.5±15.5 | 80.3±15.3 | 78.8±14.8 | 0.99(>0.05) | 0.51(>0.05) |
|  | M/M | M/m | m/m | M/M | M/m | m/m |  |  |
|  | n=93 | n=124 | n=35 | n=138 | n=207 | n=73 |  |  |
| *rs2030324* |  |  |  |  |  |  |  |  |
| VC | 84.5±18.0 | 82.8±18.4 | 83.9±20.1 | 78.7±15.8 | 80.6±15.0 | 79.9±16.2 | 0.99(>0.05) | 0.48(>0.05) |
| IM | 62.6±17.3 | 63.6±20.5 | 69.3±17.8 | 76.4±17.1 | 75.4±17.2 | 74.9±18.1 | 0.43(>0.05) | 0.18(>0.05) |
| DM | 69.9±20.3 | 71.1±20.9 | 76.9±21.6 | 86.4±15.8 | 87.7±14.4 | 83.9±14.3 | 0.52(>0.05) | 0.07(>0.05) |
| Language | 86.9±12.7 | 85.6±13.9 | 91.3±14.0 | 93.9±12.7 | 94.2±13.8 | 94.6±12.3 | 0.16(>0.05) | 0.25(>0.05) |
| Attention | 80.4±14.4 | 81.8±16.4 | 85.0±16.0 | 88.3±21.0 | 87.9±19.9 | 84.4±19.2 | 0.96(>0.05) | 0.17(>0.05) |
| Total score | 70.8±14.7 | 71.4±16.0 | 76.1±15.8 | 80.3±15.1 | 80.7±14.8 | 78.7±15.0 | 0.61(>0.05) | 0.14(>0.05) |
|  | M/M | M/m | m/m | M/M | M/m | m/m |  |  |
|  | n=130 | n=111 | n=20 | n=229 | n=177 | n=24 |  |  |
| *rs10835210* |  |  |  |  |  |  |  |  |
| VC | 84.1±18.1 | 84.5±17.6 | 76.7±24.7 | 79.2±15.2 | 80.2±15.8 | 81.6±15.8 | 0.49(>0.05) | 0.18(>0.05) |
| IM | 63.1±17.7 | 67.5±20.9 | 55.4±15.3 | 76.7±17.5 | 73.4±16.7 | 76.5±19.9 | 0.31(>0.05) | 0.03(>0.05) |
| DM | 70.4±20.8 | 75.2±20.7 | 62.7±19.1 | 86.1±15.7 | 86.4±14.0 | 85.5±17.1 | 0.07(>0.05) | 0.07(>0.05) |
| Language | 88.0±13.8 | 87.3±14.2 | 81.0±13.4 | 94.6±13.3 | 93.0±13.0 | 94.5±12.2 | 0.21(>0.05) | 0.21(>0.05) |
| Attention | 81.5±15.3 | 83.0±15.9 | 77.8±16.9 | 89.1±20.1 | 84.7±20.2 | 87.9±17.3 | 0.11(>0.05) | 0.11(>0.05) |
| Total score | 71.6±14.7 | 74.3±16.1 | 64.1±14.9 | 80.6±14.8 | 78.9±15.0 | 80.7±15.5 | 0.24(>0.05) | 0.05(>0.05) |
|  | DNFE SZ | | | Chronic SZ | | |  |  |
|  | M/M | M/m | m/m | M/M | M/m | m/m |  |  |
|  | n=57 | n=131 | n=55 | n=93 | n=124 | n=35 |  |  |
| *rs2030324* |  |  |  |  |  |  |  |  |
| VC | 81.8±19.9 | 78.4±16.4 | 71.0±12.7 | 84.5±18.0 | 82.8±18.4 | 83.9±20.1 | 0.24(>0.05) | 0.18(>0.05) |
| IM | 70.0±15.6 | 65.1±17.2 | 61.4±15.4 | 62.6±17.3 | 63.6±20.5 | 69.3±17.8 | 0.59 (>0.05) | 0.017(>0.05) |
| DM | 73.2±20.1 | 69.3±20.4 | 69.5±18.9 | 69.9±20.3 | 71.1±20.9 | 76.9±21.6 | 0.43(>0.05) | 0.41(>0.05) |
| Language | 82.9±15.8 | 74.9±18.8 | 67.9±17.7 | 86.9±14.2 | 85.6±13.9 | 91.3±14.0 | 0.04(>0.05) | <0.001(<0.05) |
| Attention | 81.3±20.6 | 73.9±19.3 | 68.2±17.8 | 80.4±14.4 | 81.8±16.4 | 85.0±16.0 | 0.25(>0.05) | 0.004(>0.05) |
| Total score | 72.5±17.0 | 66.5±15.6 | 61.1±12.7 | 70.8±14.7 | 71.4±16.0 | 76.1±15.8 | 0.24(>0.05) | <0.018(>0.05) |
|  | M/M | M/m | m/m | M/M | M/m | m/m |  |  |
|  | n=58 | n=132 | n=53 | n=229 | n=177 | n=24 |  |  |
| *rs6265* |  |  |  |  |  |  |  |  |
| VC | 77.9±18.5 | 77.2±16.5 | 77.7±16.5 | 82.0±19.5 | 84.4±18.4 | 85.9±17.2 | 0.69(>0.05) | 0.49(>0.05) |
| IM | 66.6±16.4 | 65.2±17.3 | 64.6±15.3 | 63.7±18.3 | 64.5±19.9 | 65.7±18.2 | 0.97(>0.05) | 0.50(>0.05) |
| DM | 68.2±19.7 | 70.1±20.3 | 72.8±19.6 | 71.4±21.2 | 71.9±21.0 | 73.2±20.0 | 0.48(>0.05) | 0.91(>0.05) |
| Language | 78.4±16.1 | 74.4±19.2 | 73.6±19.3 | 87.8±15.5 | 86.1±13.1 | 89.8±13.2 | 0.80(>0.05) | 0.10(>0.05) |
| Attention | 74.8±20.2 | 74.8±19.0 | 72.7±21.3 | 82.2±14.0 | 82.1±15.7 | 82.8±15.7 | 0.58(>0.05) | 0.77(>0.05) |
| Total score | 67.3±15.8 | 66.6±15.6 | 66.3±16.5 | 71.8±15.3 | 72.1±15.8 | 73.7±14.4 | 0.99(>0.05) | 0.64(>0.05) |
|  | M/M | M/m | m/m | M/M | M/m | m/m |  |  |
|  | n=158 | n=76 | n=4 | n=167 | n=84 | n=7 |  |  |
| *rs12273539* |  |  |  |  |  |  |  |  |
| VC | 77.0±16.5 | 76.7±16.1 | 102.0±34.7 | 83.7±18.5 | 84.9±18.5 | 90.4±22.5 | 0.02(>0.05) | 0.71(>0.05) |
| IM | 65.1±16.0 | 65.1±17.5 | 76.0±26.3 | 64.3±19.1 | 64.5±19.2 | 70.4±20.6 | 0.37(>0.05) | 0.77(>0.05) |
| DM | 70.9±19.9 | 67.9±19.8 | 80.3±31.0 | 70.7±20.3 | 74.4±21.4 | 76.7±25.7 | 0.58(>0.05) | 0.18(>0.05) |
| Language | 75.6±18.6 | 73.6±18.6 | 83.5±24.3 | 86.3±14.2 | 89.0±13.8 | 91.9±6.5 | 0.63(>0.05) | 0.26(>0.05) |
| Attention | 73.9±20.1 | 74.9±18.8 | 84.3±30.0 | 80.9±15.6 | 84.4±15.1 | 85.9±16.5 | 0.12(>0.05) | 0.62(>0.05) |
| Total score | 66.5±15.5 | 65.9±15.7 | 83.8±18.2 | 71.3±15.0 | 74.1±16.0 | 78.9±16.1 | 0.04(>0.05) | 0.37(>0.05) |
|  | M/M | M/m | m/m | M/M | M/m | m/m |  |  |
|  | n=126 | n=99 | n=14 | n=130 | n=111 | n=20 |  |  |
| *rs10835210* |  |  |  |  |  |  |  |  |
| VC | 76.7±17.5 | 79.3±17.0 | 71.1±13.1 | 84.1±70.1 | 84.5±17.6 | 76.7±24.7 | 0.11(>0.05) | 0.99(>0.05) |
| IM | 64.3±17.0 | 66.8±16.7 | 64.3±14.4 | 63.1±17.7 | 67.5±20.9 | 55.4±15.3 | 0.04(>0.05) | 0.12(>0.05) |
| DM | 70.0±20.3 | 71.5±19.6 | 62.4±20.4 | 70.4±20.8 | 75.2±20.7 | 63.7±19.1 | 0.03(>0.05) | 0.28(>0.05) |
| Language | 73.1±19.8 | 76.6±17.3 | 82.7±13.8 | 88.0±13.8 | 87.3±14.2 | 81.0±13.4 | 0.57(>0.05) | 0.03(>0.05) |
| Attention | 73.8±20.0 | 75.4±19.8 | 75.3±21.6 | 81.5±15.3 | 83.0±15.9 | 77.8±16.9 | 0.51(>0.05) | 0.46(>0.05) |
| Total score | 65.9±16.7 | 68.0±15.3 | 64.6±13.3 | 71.6±14.7 | 74.3±16.1 | 64.1±14.9 | 0.05(>0.05) | 0.22(>0.05) |

^a^ Values are shown as mean ± SD.

Note: IM immediate memory; DM delayed memory; VC visuospatial/constructional.
